# Small and Intermediate Calcium Activated Potassium Channels in the Heart: Role and Strategies in the Treatment of Cardiovascular Diseases

**DOI:** 10.3389/fphys.2020.590534

**Published:** 2020-11-23

**Authors:** David Weisbrod

**Affiliations:** Independent Researcher, Tel-Aviv, Israel

**Keywords:** cardiac electrophysiology, SKCa channel, arrhythmia, cardiovascular diseases, blockade and antiblockade effect, IKCa channel, atrial fibrillation, heart failure

## Abstract

Calcium-activated potassium channels are a heterogeneous family of channels that, despite their different biophysical characteristics, structures, and pharmacological signatures, play a role of transducer between the ubiquitous intracellular calcium signaling and the electric variations of the membrane. Although this family of channels was extensively described in various excitable and non-excitable tissues, an increasing amount of evidences shows their functional role in the heart. This review aims to focus on the physiological role and the contribution of the small and intermediate calcium-activated potassium channels in cardiac pathologies.

## Small Conductance Calcium Activated Potassium Channels (SK1, SK2, SK3)

### Characterization, Structure, and Functional Properties

First evidences of calcium-activated potassium channels were assessed under electrophysiological experiments in nerve cells from mollusks. In 1970, Meech and Strumwasser observed an increase in neuron permeability for potassium when intracellular concentrations of calcium chloride were experimentally raised (Meech and Strumwas, 1970; Adelman, 2016). In various gastropod models, the same team confirmed that increased intracellular Ca^2+^ concentrations were associated with hyperpolarization of the membrane potential, raising the hypothesis of a direct channel regulation by [Ca^2+^]_*i*_ (Meech, 1972). Interestingly, the effects were reduced in the presence of the calcium chelating agent EGTA (Meech, 1974). Similar associations between membrane potential voltages and intracellular [Ca^2+^] were reported in cat spinal motoneurones (Krnjević and Lisiewicz, 1972) and lately in T-lymphocytes (Tsien et al., 1982).

With the emergence of specific pharmacological tools such as apamin (Lazdunski, 1983) or charybdotoxin (Miller et al., 1985), it became possible to isolate several conductances and to study their electrophysiological properties. Apamin-sensitive current was quickly reported in several cell types such as neuroblastoma cells (Lazdunski, 1983), rat myocytes (Romey and Lazdunski, 1984), bullfrog sympathetic ganglion cells (Pennefather et al., 1985), hippocampal CA1 pyramidal neurons (Lancaster and Nicoll, 1987), or rat chromaffin cells (Neely and Lingle, 1992a, b). However, taking into consideration the differences in the cellular preparations or in the experimental conditions, numerous studies of this decade were heterogeneous in the biophysical parameters reported. Not only the kinetic of activation (Lancaster and Nicoll, 1987; Neely and Lingle, 1992a; Park, 1994; Xia et al., 1998) but also the single conductance experimentally estimated, ranging from 4 to 26 pS with symmetrical solutions (Blatz and Magleby, 1986; Mahaut-Smith and Schlichter, 1989; Lancaster et al., 1991; Grissmer et al., 1992, 1993; Partiseti et al., 1992), varied within the different reports, making it challenging to make a consensus. SK channels have relatively similar steady-state activation curves for Ca^2+^ (half activation reported around 300–700 nM) (Park, 1994; Kohler et al., 1996; Xia et al., 1998). This low affinity for calcium suggests a physiological activation of those channels mostly when intracellular calcium levels are elevated. In various excitable cells, those conductances were assumed to play a role either in the spike repolarization (Pennefather et al., 1985; Lancaster and Nicoll, 1987) or in the afterhyperpolarization (Blatz and Magleby, 1986).

The real and actual classification came in Kohler et al. (1996) could probe on cDNA hippocampal libraries and then clone and study the three SK-related mammalian members. Globally, those channels exhibit a high sequence homology (60%), especially within their transmembrane domains (80–90%). Pharmacologically, they differ by their drug sensitivity. KCNN1, the gene encoding the human SK1 channel (KCa2.1), is located on chromosome 19. The canonical predicted primary structure of SK1 is a 543 amino acids sequence, although other isoforms have been reported. For instance, C-terminal spliced variants derived channels have a reduced affinity for calmodulin binding, and their expressions seem to be tissue specific (Zhang et al., 2001). SK1 is moderately sensitive to apamin (IC_50_ 1–12 nM) (Wulff et al., 2007).

KCNN2 is located on chromosome 5 and encodes the SK2 (KCa2.2) channel. Two human isoforms of variable sizes have been currently reported (Willis et al., 2017). The shortest spliced variant, SK2 isoform b (SK2-hib), lacks the N-terminal region and the S1–S5 transmembrane domains (Willis et al., 2017). Thus, these channels exhibit the strongest affinity for apamin (IC_50_ 30–200 pM) or tamapin (Wulff et al., 2007).

Finally, the SK3 calcium-activated K^+^ channel (KCa2.3) is encoded by KCNN3, located on chromosome 1. Although different human sliced variants have been reported (Tomita et al., 2003), including the ones in the mitochondria from ventricular cardiomyocytes (Yang et al., 2017), the longest form (731 amino acids) is considered as being the canonical one. Similarly to SK1, SK3 channels are also moderately sensitive to apamin (IC_50_ 1–20 nM). Apamin sensitivity is dependent on the presence of two residues (aspartate and asparagine), oppositely located inside the deep pore of the channels and interacting with the toxin (Ishii et al., 1997).

Structurally, those channels are, overall, similar to the well-studied K^+^-voltage-dependent Kv channels. The pore-forming subunit is a tetramer formed by α-subunits. Each monomeric subunit is formed by cytoplasmic amino and carboxyl termini and by six transmembrane segments (S1–S6) (Kohler et al., 1996). The S5-P loop-S6 segments constitute the pore and potassium selectivity filter, and the S4 transmembrane domain contains two positively charged arginine amino acids. Interestingly, the number of positive charges found in the S4 TM is not sufficient to confer to the SK channels voltage dependence, a notable difference with the similar structure found in voltage-gated ion channels (Wulff et al., 2007).

The three clones contain several consensus phosphorylation target sequences, located in their cytosolic amino and carboxyl termini. The C-terminal is constituted by four alpha helices, named “helices A, B, C, and D,” respectively. Although those channels do not bind directly to calcium because of the absence of the EF-hand domain, their activity is exclusively calcium-dependent (Kohler et al., 1996). In fact, SK calcium-activated potassium channels are constitutively binding calmodulin protein (CaM), which plays the role of a Ca^2+^ sensing β-subunit. Grissmer and his colleagues already raised the hypothesis of the interaction of a single calmodulin per monomer, based on the estimated Hill coefficient of the apamin-sensitive conductance that they isolated from human T-lymphocytes (Grissmer et al., 1992).

In the absence of calcium, SK channels bind CaM through their C-terminal A-D helix, whereas in the presence of calcium, regions B–C and B–D are involved in the interaction (Xia et al., 1998). These conformation changes suggest that the constitutive SK-association to calmodulin is enhanced with calcium. More recently, structural works confirmed that each CaM protein binds the C-terminal calmodulin binding domain (CaMBD) of the channel monomer through its C-lobe domain and linker, involving electrostatic interactions (Keen et al., 1999). The EF domains of the CaM C-lobe do not bind calcium, whereas the two cytoplasmic EF domains of the N-lobe are accessible and available for such interactions. In a described chemo-mechanical gating model, the Ca^2+^ binding of the CaM would allow a change of conformation of the N-lobe and its interaction to the CaMBD from another monomer. The force created would be transduced to the S6 segment, act as a lever, and drive the SK channel opening (Schumacher et al., 2001). Additionally, the N-terminal EF domains were also shown to stabilize the SK-calmodulin interaction and the channel gating (Li W. et al., 2009). Finally, experiments of targeted mutagenesis on the CaMBD of SK2 decreased the expression of the channel on the membrane of oocytes, suggesting an involvement of calmodulin in surface expression regulation, independently to calcium (Maylie et al., 2004).

Small conductance Ca^2+^ activated K^+^ channels can be modulated by additional mechanisms. SK channels contain accessible Serine residues: one in the amino and four in the carboxyl-terminal regions, which can potentially be phosphorylated directly by protein kinase A (PKA) (Ren et al., 2006). Although PKA regulation seems to affect membrane recruitment, the exact consequences are still debated. In a transient expression system, rSK2 phosphorylation by PKA is associated with a reduction of the channel expression at the cell membrane (Ren et al., 2006). This regulation seems C-terminal specific, since SK trafficking is not altered when the three “major” Serine residues are replaced by non-phosphorable amino acids. On the other hand, PKA phosphorylation of the fourth carboxyl Serine is associated with a functional upregulation of SK2 in hypertrophic rat cardiomyocytes (Hamilton et al., 2020). In murine colonic myocytes, the open probability of SK channels is increased after phosphorylation by calmodulin kinase II (Kong et al., 2000).

### Expression and Role in the Heart

#### In the Atrial and the Ventricular Myocardium

The first reports of the existence of SK channels in the heart came recently. Xu et al. (2003) showed that a low concentration of apamin or dequalinium chloride sensibly delayed the late repolarization of the action potentials (AP) recorded in mice and in human isolated atrial cells. An apamin-sensitive current was isolated and showed an inward rectification, similarly to what was described in other tissues, and a single conductance of 3 pS was estimated. Protein expression of SK2 was confirmed across several species (mice, human, cats) and was consistently higher in atrial than in ventricular biopsies.

Two years later, the same group identified SK1 and SK3 in mouse atrial and ventricular cardiomyocytes (Tuteja et al., 2005). Quantification of the mRNA levels by qRT-PCR showed a higher expression of SK1 followed by SK2 transcripts in mouse atrial compared with ventricle cardiomyocytes. The levels of SK3 were similar within the two cell types. In their experiments, the authors confirmed that the action potential recorded from atrial cells was prolonged after pharmacological blockade of SK2 by apamin. However, the effect was amplified when BAPTA-AM, a calcium chelator, was diffused onto the inside of the cytoplasm and blocked the other SK channels expressed, confirming their involvement in the atrial late repolarization (Tuteja et al., 2005). In a mouse model with null mutation of SK2 channels, atrial late repolarization was strongly delayed compared with wild-type (WT) animals (Li N. et al., 2009). Reciprocally, the atrial repolarization and the action potential duration at 90% repolarization (APD_90_) are shortened compared to WT in mice overexpressing SK3 (Zhang et al., 2014).

In humans, SK1, 2, and 3 are expressed in atrial and ventricular cells (Xu et al., 2003; Skibsbye et al., 2014). In human atrial cells, higher transcript levels of SK2 and SK3 are observed compared with SK1. In a study that investigated the electric properties of “sinus rhythm” atrial cells isolated from the right atrial appendage of patients suffering from coronary artery or valve disorders, SK inhibition delayed the repolarization and elongated the action potential duration (APD) (Skibsbye et al., 2014). In addition, under the pacing of those cells, SK channel blockade leads to a depolarization of the resting membrane potential associated with a decrease in the AP amplitude.

The normal physiological function of the SK channels in ventricular cardiomyocytes is poorly understood and is still discussed. Pharmacological blockade of SK channels in rodents does not modify the ventricular AP and is consistent with the higher calculated IC_50_ for apamin in those cells than in atrial cells (Xu et al., 2003). Similar experiments and observations were made in canines (Bonilla et al., 2014) and humans, confirming a substantial role of these sarcolemmal conductances under physiological conditions in the ventricle (Skibsbye et al., 2014). In animal models, ventricular late repolarization is not altered in SK2 KO compared with WT mice (Li N. et al., 2009). On the other hand, it was shown that their cellular overexpression by adenovirus can shorten the rat ventricular AP (Terentyev et al., 2014).

Although SK channels were mostly thought to assemble as homotetramers, recent evidences show that SK1, SK2, and SK3 can form heterotetrameric channels through their coil–coil C-terminal domains in expression systems but also in mice or human atrial and ventricular tissues (Tuteja et al., 2010). Like their homotetrameric analogs, SK2–SK3 heterotetrameric proteins are functional channels and do play a role in atrial repolarization. Those channels lose their sensitivity for apamin, but their blockade by UCL1684 increases the “beat to beat variability” and provokes AP triangulation, in addition to the well-described delayed repolarization (Hancock et al., 2015).

SK channels can be activated by several sources of calcium—external or internal. They have been shown to colocalize with voltage-gated calcium channels in specific cellular types, such as mouse chromaffin cells (Vandael et al., 2012). Although those two channels do not physically interact, formation of microdomains, in which extracellular calcium entry activates the SK channels and regulates the firing pattern, occurs. In mouse atrial cells, SK2 colocalizes with L-type Ca^2+^ channels through α-actinin interactions, and their activation has been shown to occur essentially with external calcium. Extremely reduced SK2 currents and shortened atrial AP are observed in Cav1.3 null mice (Lu et al., 2007). In an additional mechanism, calcium can be released to the cytosol from the SR, in sparks, transients, or waves. In rat ventricular cells overexpressing SK2, enhanced or depleted SR calcium release affected the SK current recorded, independently to external calcium (Terentyev et al., 2014).

#### In the Conduction System

In addition to the working myocardium, SK channels are expressed in the conduction system. Pharmacological evidences demonstrated the existence of a small apamin-sensitive current in rabbit isolated sinoatrial (SAN) cells (Chen et al., 2013). Action potentials recorded in the presence of the SK blocker show a reversible decrease in the beating rate activity and an elongation of the APD associated with a delayed repolarization (Chen et al., 2013). SK1, SK2, and SK3 are detected at the transcript level, and the channel expressions can be seen by immunostaining in isolated mouse sinoatrial cells (Torrente et al., 2017). In agreement with what was observed in rabbit, the mouse SAN apamin-sensitive current reported in this work is small. However, in addition to what was previously reported, its blockade leads to a shallower pacemaker slope and a partial depolarization of the maximal diastolic potential (MDP) (Torrente et al., 2017).

SK2 channels are also expressed and functional in the atrioventricular node (AVN) (Zhang et al., 2008). ECG recordings from transgenic mice lacking SK2 show an elongation of the PR interval, whereas the opposite is seen when the channel is overexpressed (Zhang et al., 2008). At the cellular level, the spontaneous firing frequency of the AVN cells isolated from the null mutation mice are decreased compared with the same cells from WT or SK2 overexpressed animals. Interestingly, the MDP from the two types of mutant AV node cells remains unchanged.

#### In the Inner Organelles

On top of their role at the cellular membrane, channels can be additionally expressed at the membrane of inner organelles. In guinea pig ventricular cells, a KCa channel has been found in the mitochondria. The “mKCa” channel was purified from mitochondria membranes with standard biochemical techniques and is observed by immunostaining or by electron microscopy in the inner membrane of the mitochondria (Stowe et al., 2013). The single current recorded from purified mitochondrial channels is calcium-dependent and apamin-sensitive, similarly to the regular KCa2.2. In a model of ischemia reperfusion, Stowe et al. (2013) showed that hearts that were preconditioned with an mKCa pharmacological opener showed better LV pressure or coronary flow and a markedly reduced infarct size compared with untreated hearts. Those benefits are reduced when an O_2_^–^ dismutator is added together with the SK opener, suggesting a relation between those channels and the transient benefit of pathways related to the synthesis of superoxide radicals. In a longer term, however, the increased K^+^ flux in the mitochondria is associated with lower mitochondrial Ca^2+^, to a fine-tuned regulation of the mitochondrial energetic state, and decreased O_2_^–^ production, suggesting a protective role of those channels against cardiac injury (Stowe et al., 2013). The same group reported the existence of mitochondrial SK3 KCa isoforms in human and guinea pig ventricular cells (Yang et al., 2017). The carboxyl terminal extremity of those channels is crucial for their proper trafficking at the mitochondria membrane. The calcium from the mitochondrial matrix might activate them, resulting in the reduction of a redox state, and subsequently to cardioprotective mechanisms, similarly to other mKCa channels (Yang et al., 2017).

### Implication in Cardiopathologies

Beside their normal physiological functions, small conductance potassium channels are actively involved in the mechanism of several heart diseases.

#### Atrial Fibrillation

Atrial fibrillation (AF), the most common cardiac disorder, is often initiated by an electric dysfunction in pulmonary vein (PV) cells. Isolated PV cells from healthy rabbits do have an intrinsic small apamin-sensitive current, which physiologically plays the role of “repolarization reserve.” In the presence of apamin, the spontaneous electric activity is decreased, and the repolarization is delayed (Chen et al., 2013).

The first evidences of involvement of SK channels in atrial remodeling and AF came from experiments conducted on an isolated burst-paced atrium from rabbits (Özgen et al., 2007). This model, which simulates the pulmonary veins ectopic foci observed in AF, is associated with higher trafficking and expression of SK2 protein at the membrane of those cells and a bigger “apamin-sensitive” current. Consequently, the repolarization of the PV cells is shortened following the remodeling. ECG recorded from genetic modified mice lacking KCa2.2 channels after extrastimulation shows an AF pattern but no ventricular disorders. At the cellular level, action potential recorded from SK2 KO atrial cells shows occurrences of early afterdepolarizations (EADs) compared with WT mice atrial cells (Li N. et al., 2009). In *ex vivo* or *in vivo* WT animal models in which the atrium was acutely paced and generated induced paroxysmal AF, the duration of reversion to sinus rhythm was decreased under the infusion of SK2 blockers (Diness et al., 2010). The antiarrhythmic effect of SK blockade was associated with an elongation of the refractory period (aERP) and a termination of reentry phenomena (Diness et al., 2010; Skibsbye et al., 2011) without affecting the QT interval. Those protective effects of SK blockers on the atrium were reversible after washout, since the aERP was reduced and AF episodes were induced *de novo*. Mechanistically, the reduced SK currents would delay the repolarization of the action potentials in the atrium and the AV node, elongate the refractory period, and prevent reentrant circuits. In a canine chronic induced AFs model, hearts in which the atrium was “tachy-pached” with unipolar electrodes for 1 week underwent pathological remodeling (Qi et al., 2014). Although SK currents are bigger in the pulmonary veins compared with the left atrium in both control and paced animals, the SK open probability calculated from single channel recordings is higher in chronic AF (Qi et al., 2014). SK2 transcripts and protein expression levels were found higher in pulmonary veins but identical in diseased and healthy animals, whereas SK2.1 protein expression is specifically increased after atrial tachy-pacing (AT-P). Action potentials recorded from left atrial or PV cells have shorter duration (APD_90_) in AF animals. Consistently, the atrial effective refractory period observed in ECG is shortened in those dogs, and the AF episodes are maintained, suggesting a direct involvement of the SK currents in the pathophysiology mechanisms of chronic AF. Finally, SK blockade elongates the AP repolarization at a cellular level and improves the AF episodes by elongating the refractoriness in ECG from AF dogs (Qi et al., 2014).

In human, however, observations are different or debated and may suggest a dynamic and chronologic pathological remodeling controlled by molecular regulations. Li et al. (2011) showed that SK2 current density is bigger in atrial cells from patients diagnosed with persistent AF (at least 6 months) than in non-AF patients. At a molecular level, CAMKII levels are increased in human AF atrial cells, associated with a left shift of the SK calcium dose-response curve and bigger currents (Fan et al., 2018). Oppositely, in atrial biopsies from chronic AF patients (more than 6 months preceding a medical surgery), the SK1, SK2, (Yu et al., 2012) or SK3 (Skibsbye et al., 2014) transcripts and protein levels are reduced compared with atrial biopsies obtained from patients with sinus rhythm. Those observations are in good agreement with the work of Ling et al. (2013), which described a negative regulation of SK3 channels in chronic AF. In fact, this group reported that the micro-RNA miRNA-499, which binds the 3′UTR of KCNN3, is upregulated in human atrial cells isolated from chronic AF patients and decreases SK3 protein expression by nearly half. A GWAS conducted in 1,335 patients showed that a common SNP variant, located in the intron between the first and the second exons of KCNN3, is positively correlated to lone AF (Ellinor et al., 2010). The apamin-sensitive current is decreased in cAF-isolated cells and could explain the attenuation of the AP elongation observed when specific SK blockers are used (Yu et al., 2012; Skibsbye et al., 2014). Taken together, those data suggest a low involvement of the SK channels in chronic AF.

#### Heart Failure

Since AF and heart failure are closely related and often coexist in patients, an assumption could be that SK channels might be involved in this condition. In a canine model of recent or persistent heart failure with reduced ejection fraction (HFrEF) (1 and 4 months) with or without superimposed AF, Bonilla et al. (2014) extensively pointed out the existence of a dynamic remodeling. In paced untreated cardiomyocytes from healthy and recent heart failure (HF) animals without AF, APs are unmodified whereas those are drastically elongated four months after the HF event. Similarly, although a pharmacological blockade by apamin is ineffective in normal ventricular cells, its effects on the AP duration and the arrhythmogenicity are positively correlated to the duration of the disease (Bonilla et al., 2014).

In chronic HFrEF, the protein level of SK3—but not SK2—is strongly increased in the ventricle, whereas both proteins are overexpressed in the atrial cells. SK current density is bigger in rabbit HF ventricular cells, and an increasing gradient seems to exist between the endocardial, midmyocardial, and epicardial myocytes (Chua et al., 2011; Chang et al., 2013b). Consistently with what is shown in AF only (Qi et al., 2014), the AP of atrial cells is strongly shortened in animals with chronic HFrEF and superimposed AF. In human HFrEF patients, the same group observed an increase in the ventricular levels of SK2 and SK3 in biopsies from explanted end-stage failing hearts compared with non-HF patients (Bonilla et al., 2014). Strong beat-to-beat variability and arrhythmic patterns are visible when the HF isolated ventricular myocytes are exposed to apamin in mice, humans, and rabbits (Chang et al., 2013a; Chang and Chen, 2015). Taken together, those data suggest that SK channels are upregulated in chronic HF and are involved in the maintenance of the ventricular stability (Hsieh et al., 2013; Bonilla et al., 2014; Terentyev et al., 2014; Chang and Chen, 2015).

#### Cardiac Arrhythmia

Besides their role in AF, SK channels are associated with other pro- or antiarrhythmic phenomena. In telemetry recordings obtained from SK2 KO mice, AVN dysfunctions such as complete AV block with AV dissociation are visible (Zhang et al., 2008). Adenoviral overexpression of SK2 in WT rat ventricular cells is associated with an attenuation of induced afterdepolarizations (DADs) provoked by SR Ca^2+^ depletion in the presence of caffeine (Terentyev et al., 2014). On the other hand, DADs, severe bradycardia, advanced AV block, and higher incidence of sudden death occur in a murine model overexpressing SK3 (Mahida et al., 2014). In a rabbit failing heart, apamin suppresses the recurrent spontaneous ventricular fibrillation episodes (Chua et al., 2011). Mitochondrial SK channels may also be relevant therapeutic targets in the management of arrhythmia. Cardiac hypertrophy is associated with mitochondrial ROS synthesis, oxidative stress, and abnormal intracellular calcium handling. In addition to the effect on ROS levels and related cardioprotection, mSK activation reduces the frequency of ER spontaneous calcium waves and the occurrences of ventricular arrhythmia (Kim et al., 2017). The exact mechanism is unclear, but the reduced oxidation levels could improve the functional stability of ryanodine receptors (RyRs), thus preventing arrhythmic SR Ca^2+^ leakage and subsequent DADs.

#### Myocardial Infarction

Myocardial infarction (MI), such as heart failure, is a condition associated with an electric remodeling. Potassium currents such as the transient outward Ito, the rapid IKr, slow IKs, and a delayed rectifier are reduced (Nattel et al., 2007). In a rabbit model of chronic MI without HF, AP width and intracellular calcium transient duration are shorter in the peri-infarct zone and the remote zone compared with healthy ventricular cells, suggesting progressive remodeling (Lee et al., 2013). Isolated ventricular cells from the peri-infarct zone have a larger “apamin-sensitive” current density than cardiomyocytes from the unaffected zone, which may explain the greater elongation of the AP repolarization under SK blockade in the altered tissue. Recovery from pacing experiments shows a shortening of the AP repolarization in MI ventricle only, which is reverted with apamin. Taken together, those results suggest an extensive compensatory role of SK channels in the repolarization in a MI heart.

## Intermediate Conductance Calcium Activated Potassium Channels (SK4)

### Characterization, Structure, and Biophysical Properties

The intermediate calcium activated channel SK4 (KCa 3.1) is encoded by the gene KCNN4, located on the q13.2 part of chromosome 19 in humans (Ghanshani et al., 1998). Historically, the hypothesis of a calcium-activated potassium channel was raised by Gardos (1958) when he noticed a correlation between the potassium outflow from erythrocytes and the intracellular EDTA/calcium competition. Four decades later, SK4 has been cloned (Ishii et al., 1997; Joiner et al., 1997), biophysically characterized, and formally identified in the erythrocytes (Hoffman et al., 2003). Although the main channel is a 427 amino acids protein, different mRNA transcripts have been reported (2.1, 2,5, 3.2, and 4.5 kb), suggesting different splice variants. However, no SK4 isoforms have been currently reported. SK4 is strongly expressed in non-excitable cells such as erythrocytes, lymphocytes, placenta cells, lung, prostate, bladder, or smooth muscle cells. Electrically, its single conductance varies from 12 to 42 pS (Hamill, 1981; Grygorczyk et al., 1984; Partiseti et al., 1992; Joiner et al., 1997; von Hahn et al., 2001), and the current exhibits the same inward rectification described in other SK channels. Like other SK channels, the kinetic of the deactivation of the channel is slow and calcium-independent (Berkefeld et al., 2010). However, SK4 differs by its higher affinity for intracellular Ca^2+^ (half activation at 95 nM free Ca^2+^) (Joiner et al., 1997), compared with other SK channels (300–700 nM) (Chang and Chen, 2015). Taken together, those biochemical parameters allow SK4 channels to play a functional role at physiological basal [Ca^2+^]_*i*_ concentrations (around 100 nM) or lower and at late phases of the action potential. Pharmacologically, SK4 is insensitive to apamin but blocked by different agents such as dequalinium chloride, clotrimazole, or, more recently, TRAM-34 and senicapoc.

**FIGURE 1:**
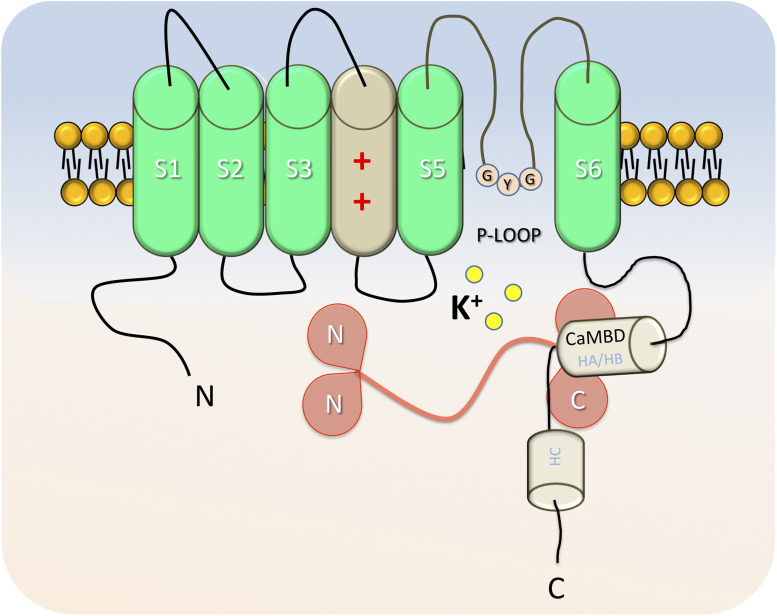
Representative cartoon of a SK4 α-subunit in absence of calcium. Each monomer is formed by six transmembrane domains (S1–S6), which are prolonged by cytoplasmic N and C termini. S4 is the voltage sensor, with positively charged amino acids. The segment (P-loop) between S5 and S6 forms the pore of SK4 and includes the GYG “selectivity filter” for potassium. The Calmodulin binding domain (HA and HB helixes), binds Calmodulin on the C-lobes. In low [Ca^2+^]_*i*_ conditions, the N-lobes of Calmodulin are free and positioned closely to the S2-S3 intracellular segment. Helix C, toward the cytosol is involved in the assembly of the α-subunits as a functional channel.

Structurally, SK4 is also very close to the canonical voltage-gated potassium channels, even if it presents a low homology (40–50%) with the other SK subfamily (Ishii et al., 1997; Joiner et al., 1997). KCa3.1 is a tetrameric protein of approximately 95 Å; in length and 120 AÅ; in width. Each monomer is formed by six transmembrane domains (S1–S6) and prolonged by cytoplasmic N and C termini (Figure 1). S4, the voltage sensor, contains two positively charged arginines, which is not enough for voltage dependence. The hydrophobic segment (P-loop) between S5 and S6 forms the pore of SK4 and contains the GYG consensus sequence, which confers to those channels a “selectivity filter” for potassium (Joiner et al., 1997). Compared with SK2 channels, the amino and carboxyl extremities are shorter, although conserved features are observed in the C-terminal (Ishii et al., 1997). The proximal part of the C terminal, previously named Ct1, is formed by the alpha helixes HA and HB, while the Ct2 distal part contains the HC helix and a Leucin zipper domain involved in inter-unit interaction (Joiner et al., 2001). Ca^2+^ binding of SK4 is indirect since no EF hands are reported in the channel (Joiner et al., 1997; Khanna et al., 1999). Instead, and similarly to what was described in SK2 (Schumacher et al., 2001), the first 62 AA of the proximal C terminal are involved in the binding of calmodulin (Fanger et al., 1999; Khanna et al., 1999). HA and HB helixes cross while forming the calmodulin binding domain (CaMBD), a pocket parallel to the cell membrane, which preassociates with the C-Lobe of calmodulin in a calcium-independent manner. Four calmodulins interact with the channel and bind Ca^2+^ through the EF motif of their N-lobe (Schumacher et al., 2001). Mutations in calmodulin or in the CaMBD strongly decrease the IKCa current in various models, suggesting a role of calmodulin in the gating and trafficking of the channel (Fanger et al., 1999; Joiner et al., 2001). In addition, mutations in the leucin zipper also lead to drastic reduction of the current and membrane protein expression (Khanna et al., 1999), confirming a role of the distal C-terminal in the proper folding and trafficking of SK4 independently to calmodulin. Recently, Lee and MacKinnon (2018) described in depth the structure and mechanism of the human SK4 interaction and CaM using the Cryo-EM technique. The HC helix is pointing toward the cytosol and is associated with similar helixes from the other subunits in a coil-coiled structure, essential for the proper assembly and trafficking of the channel. In the absence of calcium, the CaM N-lobe is free, oriented near the bottom of the S2 subunit, and the valine amino acids from the S6 transmembrane segment of each monomer close the gate responsible for K^+^ outflow. In the presence of calcium, the N-lobe of the CaM moves toward and binds the S4–S5 linker of an adjacent subunit, pulling it toward the cytosol. This movement leads to conformational changes in the S6 segments, enlargement of the gate, and opening of the channel. Ca^2+^–CaM interactions induce cooperative conformation changes necessary for the opening of the channel (Fanger et al., 1999).

Although nucleotides or cyclic nucleotides such as cAMP do not directly bind or modulate the channel activity (Pellegrino and Pellegrini, 1998; Gerlach et al., 2000), KCa3.1 presents potential phosphorylation sites for different kinases (Joiner et al., 1997; Gerlach et al., 2000). In the distal C-terminal part of the channel, phosphorylation consensus sites such as Serine 334 for PKA or four other amino acids for PKC have been reported. Several studies pointed out the positive regulatory role of hydrolyzed ATP on SK4 (Gerlach et al., 2000; von Hahn et al., 2001; Hayashi et al., 2004). However, the downstream mechanisms or effects are debated and seem to be species dependent. In human erythrocytes (Pellegrino and Pellegrini, 1998) and rat acinar cells (Hayashi et al., 2004), cAMP-PKA increases KCa3.1 activity, whereas controverted results have been reported in oocytes (Gerlach et al., 2000; von Hahn et al., 2001). In rat glial cells or transfected cells expressing the human SK4, PKA phosphorylation was specific to the consensus site and associated with a decrease in the current (Wong and Schlichter, 2014). Surprisingly, mutagenesis studies show that ATP-dependent regulation still occurs when consensus phosphorylation sites are altered (Gerlach et al., 2001; von Hahn et al., 2001), raising the hypothesis of alternative phosphorylation sites or other kinases involved.

PKC activators reduce the opening frequency of IKCa but not its single conductance in human erythrocytes (Del Carlo et al., 2003), whereas similar regulations have not been reported in human colon T84 cell line (Devor and Frizzell, 1998). Other kinases such as the nucleoside diphosphate kinase B (NDPK-B) (Srivastava et al., 2006) or the CaMKII (Khanna et al., 1999; Gerlach et al., 2000) regulate IKCa in T-cells and could play a role in cell activation (Srivastava et al., 2006; Wulff et al., 2007). Other factors or regulators can modulate IKCa. For instance, arachidonic acid binds the pore region of the SK4 channel and strongly decreases its current (Devor and Frizzell, 1998; Hamilton et al., 2003). Temperature can affect the open probability of SK4 and the net potassium efflux in human erythroblasts (Hoffman et al., 2003). Finally, beta-blockers can fine-tune KCa3.1 activity. In human erythrocytes, where beta-receptors are absent, submillimolar concentrations of propanolol increase the calcium sensibility of SK4, whereas millimolar concentrations block the conductance (Schwarz et al., 1989).

### Expression and Role in the Heart

The first reports that characterized SK4 in several tissues failed to detect a transcript in heart when 3′UTR or other cDNA sequences were used as a probe in Northern Blot experiments (Ishii et al., 1997; Joiner et al., 1997; Hoffman et al., 2003). With the emergence of high-throughput screening techniques, very detailed mapping of ionic channel expressions has been collected in atrial, ventricular working cardiomyocytes or conduction tissues (Marionneau et al., 2005; Gaborit et al., 2007). Surprisingly, while the expression of SK1-3 has been investigated, SK4 was overlooked.

First associations between KCa3.1 and the heart came in embryonic stem cells when Kleger et al. (2010) cultured mice and human (Müller et al., 2011) embryonic stem cells in the presence of the SK4 opener 1-EBIO and observed an enriched differentiation into cardiomyocytes, especially toward a pacemaker-like phenotype. In fact, typical “pacemaker signature genes” such as Tbx3, HCN4, or Cx30 were upregulated, whereas the MLC2V and Cx43 ventricular genes were downregulated. Inversely, cell culture in the presence of clotrimazole—but not apamin—or the elaboration of stable knocked down ES lines with shRNA against SK4 specifically decreased cardiogenesis. Recently, an independent group showed that rat adipocyte stem cells were effectively differentiated into pacemaker-like cells following SK4 adenoviral transduction. When those modified stem cells were injected in the heart, they generated an ectopic pacemaker after differentiation (Yang et al., 2019). An additional study focused on the dynamic gene expression in mouse AVN during development and reported a ninefold increase in SK4 transcripts (Horsthuis et al., 2009).

Taken together, those reports show a molecular involvement of KCa3.1 in cardiac and pacemaker fate through the activation of the ERK1/2 signaling pathway (Kleger et al., 2010; Yang et al., 2019). However, the channel expression itself and its role in the cardiac pacemaker mechanism were not mentioned.

**FIGURE 2:**
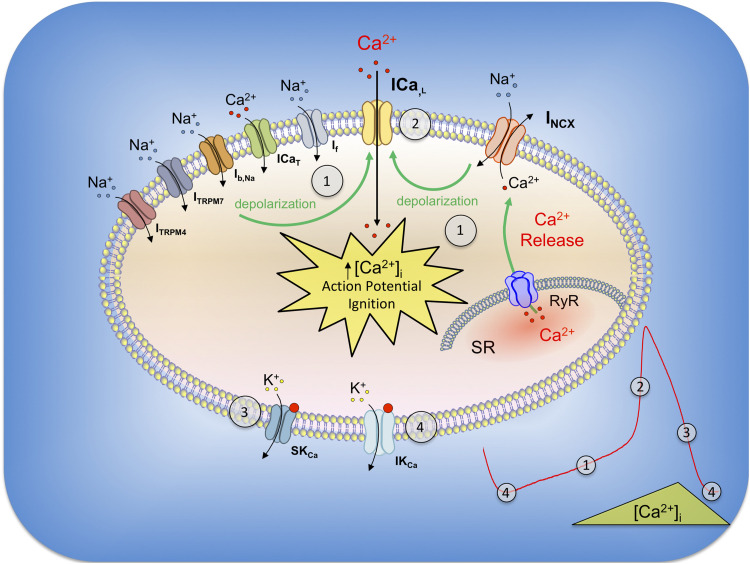
General model of the pacemaker currents in SAN and their relation with SK and IK channels. During the late repolarization of the previous action potential, SK4 are still opened and their outward currents (4) act like a MDP driving force (4), which concurrently activates HCN channels (I_*f*_ current). The gradual depolarization recruits other conductances such as Ib, Na, ICa_*T*_ (3.1 and 3.2), ITRPM4 and ITRPM7, which, altogether in synergy, are responsible for the diastolic depolarization (1). In parallel, diastolic sarcoplasmic spontaneous calcium release through RyR receptors increases the cytoplasmic level of calcium and activates the NCX-1 exchanger in its forward mode (1). The progressive Ca^2+^ extrusion operated by NCX from early to late DD gradually reduces IKCa and allows inward currents to take over. INCX, together with the other described currents, depolarize the membrane until the threshold of activation for L-type Ca^2+^ channels (ICa,L) and AP ignition (2). During the early repolarization, high [Ca^2+^]i open small SK channels (3) which contribute to this phase of the action potential. The progressive cytosolic decrease of calcium (SR reuptake and extrusion) decreases SK currents at the exception of IKCa due to SK4 higher affinity for Ca^2+^ (4). N.B. BKCa, Kv and other conductances have not been included in this cartoon for readability.

First direct evidences of the protein expression of the SK4 channel in cardiomyocytes and its physiological role were assessed in cardiomyocytes derived from human embryonic stem cells (hESC-CMs) (Weisbrod et al., 2013). Young hESC-CMs beat spontaneously, mimic the features observed in the primitive heart during development, and have been used as a model to better understand the debated mechanisms underlying the pacemaker activity (Lakatta and DiFrancesco, 2009). The diastolic depolarization (DD) or “pacemaker depolarization” results from a small net inward current across the cell membrane and is the key feature of the cardiac automaticity. It occurs during the diastole, at the end of an action potential, and is responsible for triggering the next one. Different models such as the “membrane clock” involving mainly HCN and other ion channels (Brown and Difrancesco, 1980; Hagiwara et al., 1988, 1992; Mangoni et al., 2003; Stieber et al., 2004; Demion et al., 2007; Baruscotti et al., 2010; Mesirca et al., 2014), the “Ca^2+^ clock” with its spontaneous Ca^2+^ release from the SR and subsequent NCX-1 activation (Fabiato and Fabiato, 1972; Bassani et al., 1997; Bogdanov et al., 2001; Vinogradova et al., 2004; Maltsev et al., 2006; Maltsev and Lakatta, 2007; Groenke et al., 2013), or the coexistence of both could explain this mechanism (Figure 2) (Gao et al., 2010; Lakatta et al., 2010; Zahanich et al., 2011; Billman, 2012). Blocking the If or INCX inward conductances led to a counterintuitive reversible depolarizing drift of the MDP, suggesting a mechanistic convergence of both models to a previously undescribed blocked outward current (Weisbrod et al., 2013). RT-PCR, Western blots, immunostaining, and electrophysiological characterization of the unitary conductance isolated from membrane patches of hESC-CMs identified for the first time in human-derived cardiac pacemaker cells this current as KCa1.3, the intermediate Ca^2+^ activated-potassium channel (IKCa/SK4). Under physiological conditions, pharmacological experiments with different IKCa antagonists led to a reduction of the diastolic pacemaker slope, a depolarizing drift of the membrane resting potential, and, ultimately, a cessation of the pacing in young healthy hESC-CMs (Weisbrod et al., 2013). Interestingly, the depolarized MDP but the unaffected AP duration under SK4 blockade suggests an involvement of KCa3.1 in the late repolarization, as an outward current that dynamically balances the inward pacemaker conductances in a [Ca^2+^]_*i*_-dependent manner (Weisbrod et al., 2013, 2016). In a recent study, SK4 and HCN2 ventricular adenoviral overexpressions were sufficient to increase the *ex vivo* heart frequency recorded in a complete rate heart block rat, and transform those cells into active pacemakers (Zhao et al., 2019). After a prolonged *in vitro* maturation, hESC-CMs decrease their spontaneous firing rate and lose their intrinsic pacemaker activity, similarly to the working cardiomyocytes of the primitive heart (Satin et al., 2004; Couette et al., 2006; Bakker et al., 2010). Although SK4 is still expressed in those late-stage cells, the decrease in the beating rate associated with IKCa blockade was restricted to cells that exhibited strong pacing, suggesting an important role of SK4 in pacemaker tissue (Weisbrod et al., 2013). In a mouse adult heart, the protein expression of SK4 has been shown in atrial appendages, ventricles, and also in the sinoatrial node (Haron-Khun et al., 2017). In rats, SK4 is strongly expressed in the SAN, AVN, and ventricles, but, in a less extent, in the atria (Zhao et al., 2019). In contrast, the canine protein expression of KCa3.1 is found in the atria only, suggesting interspecies variability (Yang et al., 2020a,b). Finally, the expression of KCa3.1 has been reported in endogenous cardiac progenitor cells (Neco et al., 2012).

In biopsies from adult human hearts, although SK4 is detected at the mRNA level in right atrial and ventricular tissues, the protein is expressed only in the atrium (Weisbrod et al., 2016). In SAN cells obtained from healthy mice, a TRAM-sensitive current is pharmacologically isolated under voltage clamp configuration. The spontaneous AP frequency and the pacemaker slope are strongly reduced in a reversible manner when single SAN cells are exposed to SK4 blockers alone or together with a β-agonist (Haron-Khun et al., 2017). *In vivo* telemetric recording experiments performed on WT mice show a reduction of the heartbeats and a PR elongation at rest and during treadmill exercises when SK4 blockers are injected in IP. From a mechanistic point of view, the reduction of the heartbeat and the decrease in the spontaneous firing rate of the cardiac pacemaker cells following IKCa inhibition could be explained by the reduced driving force at the beginning of the diastole. The diminution of the outward IKCa K^+^ current under SK4 blockade would impair the fine-tuning of the depolarizing currents (especially I-funny), shallow the diastolic slope, delay the time between two action potentials, and subsequently decrease the beating rate. Additionally, the depolarization of the MDP would reduce the depolarizing currents (inactivation of a subpopulation of the INa^+^ plus diminution of ICa^2+^) and the upstroke velocity (dV/dt) of the action potentials.

Taking altogether, different data show an important involvement of the intermediate calcium-activated K^+^ channel in cardiac automaticity.

### Implication in Cardiac Disorders and Arrhythmia

Scientific evidences supporting the involvement of an SK4 channel in cardiopathies are still limited, and the mechanisms are poorly understood.

In an acute model of myocardial infarct in rats, Saito and colleagues observed an increased expression of SK4 transcripts following the coronary ligation (Vigneault et al., 2018). Interestingly, the overexpression was higher in the animal group that underwent a reperfusion compared with those animals with permanent occlusion. While the related mechanism is not clear, SK4 channels might be involved in the vascular remodeling following MI since those are known to be associated with cell proliferation (Pena et al., 2000; Saito et al., 2002).

Catecholaminergic polymorphic ventricular tachycardia (CPVT) is a stress-induced ventricular arrhythmia associated with cytoplasmic calcium leakage due to mutations in calsequestrin 2 (CASQ2) (Postma et al., 2002; Wang et al., 2005), ryanodine receptor (RyR2) (Laitinen et al., 2001; Eldar et al., 2003), triadin (Priori et al., 2001), or calmodulin (Roux-Buisson et al., 2012). At rest, ECG is normal, although sinus bradycardia has been observed in patients (Wang et al., 2005). Under stress or exercise, the Ca^2+^_*i*_ overload activates NCX-1, which generates at the cellular level DADs leading to ventricle potentially fatal disorders such as ventricular premature complexes (VPCs) or non-sustained (NSVT) or sustained ventricular tachycardia (SVT) visible at ECG. In addition, CPVT is associated with SAN dysfunction caused by altered calcium homeostasis under beta-adrenergic stimulation (Makita et al., 2014; Haron-Khun et al., 2017). In cardiomyocytes derived from human induced pluripotent cells (hiPS-CMs) from CPVT2 patients carrying a CASQ2 mutation, Haron-Khun et al. (2017) showed that isoproterenol-induced DADs were neutralized in the presence of TRAM-34. Similar effects were observed in isolated SAN cells from CASQ2 KI mice expressing the same patient mutation, and a TRAM-sensitive current was isolated. Abnormal Ca^2+^ transients recorded from isolated CPVT KI mice SAN were drastically improved in the presence of the SK4 blocker. Finally, in addition to the heart rate decrease and the PR elongation, ECG arrhythmic features visible at rest and during treadmill exercises were strongly reduced or normalized when SK4 blockers were injected in IP to CPVT mice (Haron-Khun et al., 2017).

Arrhythmogenic right ventricular cardiomyopathy (ARVC) is a rare but severe cardiac condition frequently associated with mutations of proteins involved in desmosomes (Marcus et al., 2013) (Plakophilin-2, Desmoplakin, Desmoglein-2, and Desmocollin-2), structures involved in the cell–cell interactions. Ventricular cardiomyocytes progressively detach and are replaced by a fibrotic and fat tissue upon an inflammatory remodeling, leading subsequently to ventricular tachyarrhythmia and sudden death (Corrado et al., 2019). In hiPS-CMs derived from an ARVC patient carrying a mutation in the DSG2 gene coding the desmoglein-2 protein, Buljubasic et al. (2020) recently showed that protein expression of SK4 is strongly increased compared with hiPSC-CMs obtained from healthy donors. Interestingly, the transcript levels and protein expression of NDPK-B, kinase known to upregulate the IKCa current, are also increased in the lysate from ARVC cells. Consistently with this observation, the pharmacologically isolated calcium-dependent IKCa current was stronger in ARVC hiPSC cells. Quantified arrhythmic features and spontaneous pacing were higher in AP from ARVC cells compared with healthy donors (Buljubasic et al., 2020). However, intracellular addition of recombinant NDPK-D increased the IKCa current, the spontaneous beating rate, and also the frequency of arrhythmic events in the AP recorded from both healthy and ARVC cells. The reversible nature of those phenomena by exposure to protein histidine phosphatase 1 (PHP-1) points a dual regulation of SK4 in this disease. TRAM-34 perfused in ARVC hiPSC decreased the number of DADs and other arrhythmic features before depolarizing the MDP until a rapid AP cessation. Taken together, those data strengthen the pro-arrhythmic role of the SK4 upregulation through a NDPK-D-dependent mechanism and a potential approach to treat ARVC.

In addition to ventricular arrhythmia, new evidences pointed the involvement of SK4 in AF and its blockade as a potential new therapeutic strategy. In dogs, which were subjected to rapid atrial pacing for 7 h to induce AF, protein expression of SK4 expression from right and left atriums was increased compared with non-paced dogs, confirming the consistent reported proarrhythmic potential role of SK4 (Haron-Khun et al., 2017; Buljubasic et al., 2020). Infusion with 10 mg/kg TRAM-34 suppressed the AF induction in the treated group (Yang et al., 2020b). The same group confirmed the involvement of SK4 in AF and the benefit of its blockade in dogs that developed AF after acute stroke (Yang et al., 2020a). In a canine model of chronic HF, TRAM-sensitive current, SK4 transcripts, and protein expression levels are reduced compared with healthy animals (Neco et al., 2012), suggesting a pathological downregulation. The lower IKCa current density was associated with a depolarization of the cell membrane resting potential, in consistency with data observed after pharmacological blockade in previous reports (Weisbrod et al., 2013, 2016).

## Discussion

Since almost four decades, a better understanding of general pathophysiological mechanisms, an intensive management of patient lifestyle, and the development of new class of molecules led to a successful worldwide decline in mortality from major CV diseases such as coronary arterial diseases, stroke, MI, and ischemic cardiopathies (Mensah et al., 2017; Cheng et al., 2018). However, despite the major advances in cardiovascular pharmacology, disease registries show that the management of cardiac arrhythmia and HF did not benefit from this mortality reduction (Cheng et al., 2018).

With the aging of the worldwide population, the increased prevalence and costs related to those diseases, and the longer period of medical care, it is fundamental to improve the quality of treatment by providing alternative or additional therapeutic strategies.

Within the last 20 years, a growing number of evidences point out not only the physiological role of calcium-activated potassium channels in the heart but also their involvement in various cardiac disorders.

SK channels and IK channel (SK4) have been found in the atrial, ventricle myocardium and in conducting tissue from different species, including humans. Because of their low Ca^2+^ affinity (half activation at 300–700 nM), small-conductance Ca^2+^-activated K^+^ channels are likely to be involved in repolarization phases, where the cytosolic calcium is still elevated. This is well illustrated by the APD elongation reported especially in atrial cells when those are exposed to apamin. In the ventricle, on the other hand, small calcium-activated channels are less involved in the repolarization itself. They can enhance the cell stability by playing the function of “repolarizing reserve,” especially in diseases leading to an electric remodeling such as HF or MI. In contrast, intermediate-conductance Ca^2+^-activated K^+^ channels, with their higher affinity for calcium, open at physiological [Ca^2+^]_*i*_ concentrations and are involved in the late repolarization (MDP). The fact that SK4 blockade does not elongate the APD, leads to a reduction of the diastolic slope (DD Slope) and most importantly, to a depolarization of the MDP in cardiac cells with spontaneous electric activity, strengthen its role of “fine tuner” of the well-established “pacemaker inward currents” (Weisbrod et al., 2013, 2016).

In SAN cells, SKCa and IKCa blockade leads to a reduction of the pacing. Effects on the pacemaker diastolic slope or the MDP are consistent with the SK4 blockade but seem species-dependent in the case of SK1–3.

Slowing down the heartbeat is a gold-standard approach used in modern cardiology as primary therapy and as a secondary prevention. Beta-blockers are the corner stone of this strategy in the management of acute MI, HF, AF, and other arrhythmia, since those pathologies are interconnected. After treatment initiation, up-titration to optimal dosage should occur within a short time frame (“start low, aim high”) for sustainable benefits. However, in addition to the late dosage escalation or underdosing of those molecules compared with theoretical optimal treatment, the “adrenergic-escape phenomenon,” in which abnormally high concentrations of catecholaminergic hormones are produced, despite the stable β-blocker treatment, drastically worsens patient survival prognostics (Coumel et al., 1984; Frankenstein et al., 2009). Although the phenomenon has been described in very few scientific papers, its prevalence, estimated in one third of chronic HF patients, is associated with a 60% increase in mortality at 3 years compared with patients without this condition (Frankenstein et al., 2009). Other bradycardic agents such as ivabradine, which selectively blocks the I-funny (If) current, have been developed and are used currently in the management of HF and chronic stable angina (Koruth et al., 2017). However, ivabradine, similarly to β-blockers, requires dosing adjustments and has limited benefits mainly due to its specific effect on the PP interval elongation. A recent study based on an animal model of CASQ2 CPVT has shown that the decrease in the sinus rhythm provoked by ivabradine was not sufficient to improve the arrhythmic features observable with telemetric ECG recordings (Bueno-Levy et al., 2020). However, the same publication showed that SK4 blockade attenuates arrhythmic features, probably due to the AVN blockade and subsequent PR segment elongation in addition to the effect on the SAN and the PP interval. Similarly, SK2 blockade is also associated with a decrease in the heart rate and a PR segment elongation (Torrente et al., 2017).

Slowing down the heart rate and increasing the refractive period are mechanistic strategies used to prevent the reentry phenomenon and arrhythmia in the atria and the ventricle.

**FIGURE 3:**
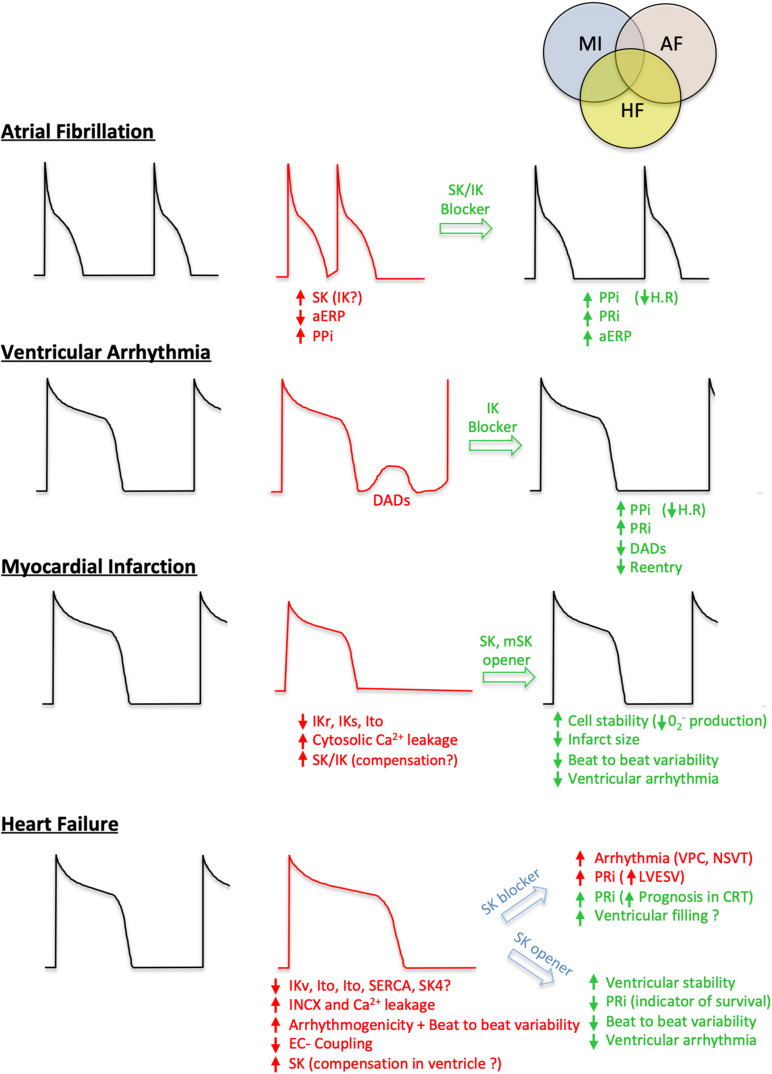
Involvement of IK and SK in cardiovascular diseases and potential therapeutic strategies. For each condition (early stage Atrial Fibrillation, Ventricular arrhythmia, Myocardial Infarction and Heart Failure), a healthy action potential is shown (black cartoon) in comparison with the altered one (red cartoon). The different positive (green) or negative (red) effects based on ISKCa and IKCa modulations are summarized. PPi, P wave Interval; aERP, atrial effective refractory period; PRi, P-R segment; H.R, heart rate; DADs, delayed after depolarization; VPC, ventricular premature contraction; NSVT, non-sustained ventricular tachycardia; LVESV, left ventricular end-systolic volume; CRT, cardiac resynchronization therapy; IKr, rapid activating delayed rectifier potassium current; IKs, slow activating delayed rectifier potassium current; Ito, voltage-gated transient outward potassium current; INCX, sodium-calcium exchanger current.

AF is the most common diagnosed cardiac arrhythmia, with an estimated actual prevalence of 37.5 million cases worldwide and a projected increase by 60% until 2050 (Lippi et al., 2020). The task force for the management of AF in Europe estimates that 25% of middle-age adults in Europe and the United States will develop AF during their lifetime (Kirchhof et al., 2016). AF management alone represents around 2% of the healthcare expenditures in European countries. Current pharmacological management is based on oral anticoagulation therapies, β-blockers, calcium channel blockers, cardiac glycosides, and Amiodarone, if patients do not reach the optimal heart rate control (Kirchhof et al., 2016). Although there is a broad therapeutic arsenal available, control of AF is not always optimal, and recurrent episodes with new ectopic foci can develop. Catheter ablation, a second-line therapy, is considered to be more effective than a pharmacological approach for persistent AF (ATTEST Trial). However, this technique requests several surgical interventions and can be associated in some cases with severe adverse events such as atrioesophageal fistulas. AF is associated with a dynamic remodeling of the small Ca^2+^-activated K^+^ currents. Initially, those currents are increased in human atrial or acute animal models and are responsible for a shortening of the APD. Pharmacological blockade of SK channels slows down the AVN conduction, increases the refractory period, decreases the reentry, and improves the reversion time from AF episodes to sinus rhythm (Diness et al., 2010; Skibsbye et al., 2011). In human chronic AF, however, downregulation of the channels prevents such an approach. Taken together, those data point to the benefits of small KCa blockade as a potential therapeutic strategy in early AF (Figure 3). Although the involvement and regulation of SK4 in AF have not been studied in depth yet, a full suppression of arrhythmic features in a canine model of acute AF has been reported after IV infusion of the SK4 channel blocker (Yang et al., 2020a,b). Further studies on models or samples from AF patients could help investigate the viability of this theory, especially in cAF.

Management of ventricular arrhythmia is complex since the modulation of a specific channel can have antiarrhythmic or proarrhythmic consequences. Genetic ablation of SK2 channels leads to an extensive PR elongation, AVN dysfunction, and AV dissociation (Zhang et al., 2008). On the other hand, similar features and severe bradycardia have been described when SK3 is overexpressed (Mahida, 2014; Mahida et al., 2014). At the cellular level, pharmacological blockade of SK4 channels has been reported to improve arrhythmic features in CPVT and ARVC, two distinct ventricular disorders. *In vivo*, IP injection of the SK4 blocker reduced the occurrence of SVT, NSVT, or VPC visible at ECG in CPVT mice in addition to a negative chronotropic effect and an elongation of the PRi (Haron-Khun et al., 2017). Thus, a subtle PR segment elongation based on a mild pharmacological AVN blockade could be beneficial in the management of cardiac arrhythmia.

Heart failure management remains the last big challenge in modern cardiology since this syndrome, based on clinical manifestations, can result from extremely various pathophysiological mechanisms. HFrEF, frequently described as a “systolic HF” in older classification, is often associated with an inflammatory state, a ventricular myocardium loss (LV dilatation), and subsequent reduction of the cardiac output and ejection fraction (EF). Inversely, HFpEF or “diastolic HF” affects the ventricular stiffness and arterial compliance, and increases the LV wall thickness and filling pressure. Recently, a “mid-range” HF (HFmrEF) has been added to the actual classification for patients with mild systolic dysfunction and diastolic dysfunction (Ponikowski et al., 2016). HF, also called the “cardiovascular cancer,” remains as malignant as prostate or breast cancers, with an estimated survival rate of 40–50% in 5 years following diagnosis (Mamas et al., 2017). The condition is progressive, associated with episodes of cardiac decompensation (acute exacerbations), which irreversibly worsens the myocardial function and patients’ quality of life. Initial pharmacological treatment is usually based on a therapeutic triad (β-blockers, ACEi or ARBi, MRA, and diuretic for decongestion), but other molecules such as ivabradine (Swedberg et al., 2010), ARNi (McMurray et al., 2014), and very recently SGLT2i (McMurray et al., 2019) have been added to the armamentarium. Most of those molecules are used in all types of HF, although they did not constantly show a benefit besides HFrEF (Koruth et al., 2017; Solomon et al., 2019), which opens the door to additional approaches.

At a cellular level, HF is associated with an electric remodeling. Na^+^ currents are increased, while Ito, IKs, and other rectifier potassium currents are reduced (Näbauer and Kääb, 1998; Nattel et al., 2007). The increased RyR Ca^2+^-leakage, reduced SERCA activity, and INCX upregulation contribute to the altered EC coupling and reduction of the contractile function (Reuter et al., 2004; Roos et al., 2007; Ottolia et al., 2013). The gain of function of SK channels, a consequence of the higher protein expression and cytosolic Ca^2+^ leakage, could possibly be a compensatory mechanism for the loss of repolarizing currents in chronic HF. Beat to beat variability and arrhythmic features such as torsades de pointe or VPC have been described in human end-stage HFrEF and animal models when SK are blocked. In congestive HF, abnormal elongation of the human PR segment (>200 ms) is associated with higher LV end-diastolic, end-systolic volumes and higher recurrences of events (McManus et al., 2009; Husby et al., 2015) and is commonly used as a marker to evaluate the impaired exercise ability of patients (Stępniewski et al., 2017). In contrast, in end-stage HF, PRi elongation is associated with better survival prognosis in patients who underwent cardiac resynchronization therapy (Kutyifa et al., 2014). Mechanistically, a delayed conduction in the AVN could have a positively influence on ventricle filling. Overall, although modulations of calcium-activated K^+^ channels could have potential positive outcomes on pacing stability or PR duration, further investigations are necessary to specify HF patient subpopulations who could positively benefit from those effects. Similarly to the remodeling seen in HF, membrane SK, mitoSK, and IK channels are upregulated in MI, especially after reperfusion. Although the mechanisms need to be elucidated, membrane IK and SK channels seem to play a role in pacing stability by counterbalancing the downregulation of other potassium currents (Lee et al., 2013). Furthermore, positive pharmacological modulation of mitochondrial SK activation decreases ROS synthesis, which improves RyR stability and reduces Ca^2+^ SR leakage and associated ventricular arrhythmia in hypertrophic hearts (Stowe et al., 2013; Kim et al., 2017; Yang et al., 2017). Finally, a preventive treatment of mSKCa reduces the infarct size in the model of ischemic perfusion (Stowe et al., 2013). Based on those cardioprotective properties, development of openers could prevent arrhythmia associated with MI.

The growing number of evidences supporting the involvement of calcium-activated potassium channels in heart diseases confirms that those players should clearly gain more consideration as potential therapeutic targets. The dual effect on the heart rhythm and the PRi associated with the blockade of KCa conductances can be beneficial in atrial or ventricular arrhythmia but could worsen other conditions such as HF or MI. Further investigations are required to better understand mechanisms and the realism of those modulations in daily care since those pathologies are interconnected and often codiagnosed in patients.

## Author Contributions

DW wrote the manuscript and prepared the figures.

## Conflict of Interest

The author declares that the research was conducted in the absence of any commercial or financial relationships that could be construed as a potential conflict of interest.

## Glossary

ACEi, inhibitors of the angiotensin converting enzyme (ACE), which prevent the formation of angiotensin II and associated vasoconstriction; AF, atrial fibrillation; AP, action potential. This is the unit pattern of the cellular electric activity; APD, action potential duration (in milliseconds). Time interval between the beginning (threshold) of an action potential and its end (hyperpolarization); APD_50,_ action potential duration at 50% of the repolarization (in milliseconds); APD_90_, action potential duration at 90% of the repolarization (in milliseconds); ARBi, inhibitors of the angiotensin II receptors. Medications used as an additional approach to prevent the vasoconstriction induced by angiotensin II; ARNi, medications used in HF, based on a combination between an angiotensin receptor inhibitor and a neprilysin inhibitor; ARVC, arrhythmogenic right ventricular cardiomyopathy; AVN (or AV node), atrioventricular node. Secondary heart pacemaker structure, responsible for a delay of the electric conduction between the SAN and the ventricles and a better blood filling; CaM, calmodulin or calcium-modulated protein. It binds Ca^2+^ and modulates ion channels properties; CaMBD, calmodulin binding domain. Structure of the ion channels that binds CaM; CAMKII, Ca^2+^-calmodulin-dependent protein kinase II; cAMP, cyclic adenosine monophosphate; CPVT, catecholaminergic polymorphic ventricular tachycardia; DD slope (pacemaker slope), diastolic depolarization slope. Initial portion of the AP caused by the involvement of several ion channels and transporters. Those proteins lead a depolarization of the membrane (slope), until the threshold for the action potential ignition; hESC-CMs, human embryonic stem-cells derived cardiomyocytes. Cardiomyocytes differentiated from human embryonic stem cells; PP interval (PPi), interval between two successive P-waves, measured in ECG (in milliseconds). P-waves reflect the SAN activity and the PPi is used to evaluate the heart rate; PKA, cAMP-dependent protein kinase; PRi (or PR segment), interval between the P-wave and the R-wave of the QRS complex, measured in ECG (in milliseconds). It evaluates the delay of propagation of the electric activity between the SAN and the ventricular myocardium; HF, heart failure; HFpEF, heart failure with preserved ejection fraction. Mostly associated with a hypertrophic heart and a reduction of its stiffness; HFrEF, heart failure with reduced ejection fraction. Mostly associated with a reduction of the ventricular myocardium mass; If or “I-funny”, depolarizing current induced by the HCN channels. Also called “pacemaker current,” it plays an important role in the SAN and the AVN during the diastolic slope of the action potential; Ivabradine, selective blocker of the HCN channel; MDP, maximum diastolic potential. Value of the membrane potential at the most hyperpolarized part of the action potential (in millivolts); MI, myocardial infarction; mSKCa, mitochondrial isoform of the SKCa channel; NCX-1, sodium–calcium exchanger-1. Electrogenic antiporter (3 sodium ions for 1 calcium) strongly involved in ventricular excitation–contraction coupling and in the pacemaker mechanism; NSVT, non-sustained ventricular tachycardia. Transient VT episodes; SAN (or SA node), sinoatrial node. Structure located in the right atrium, primary cardiac pacemaker. SERCA, sarco/endoplasmic reticulum Ca^2+^-ATPase. Pump responsible for the Ca^2+^ reuptake from the cytosol to the endoplasmic reticulum; SGLT2i, inhibitors of the sodium-glucose co-transporter-2; SR (or ER), sarcoplasmic (endoplasmic) reticulum. Organelle, which stores the cellular Ca^2+^; ROS, reactive oxygen species; RyR, ryanodine receptors. Receptors on the SR, responsible for the intracellular Ca^2+^ release; TM, transmembrane domain; TRAM-34, 1-[(2-Chlorophenyl)diphenylmethyl]-1*H*-pyrazole. SK4 blocker; VPC, ventricular premature complexes. Arrhythmia usually ectopically induced by the Purkinje fibers; VT, ventricular tachycardia.
